# On the Relation of the Teeth and Mouth to Mental Development

**Published:** 1872-06

**Authors:** Langdon Down

**Affiliations:** Physician of London Hospital, late Resident Physician of Erlswood Asylum, &c.


### SELECTED ARTICLES.

### ARTICLE IV.

### *On the Relation of the Teeth and Mouth to Mental Development.*

BY DR. LANGDON DOWN,

Physician of London Hospital, late Resident Physician of Erlswood Asylum, &c.

* * * * A year or two since a very intelligent medical practitioner in the country was called in to treat a case of infantile convulsions. The condition of the child was desperate. He poured from an ewer a stream of cold water over the occiput of the child; the convulsions ceased, the patient was rescued from impending death, but grew up to be an idiot. The friends of the child took up a position which involved a trial in a court of law, equivalent to an action for malpraxis against the medical man. By a judge's order, I, with other medical men, saw the child; and we were able to say not only that the child was an idiot then, but, by an examination of the mouth, to assert that the idiocity was embryonic as to date, that the convulsion was an epiphenomenon, and that the medical man was in no way responsible for the mental condition of the child whose life he had rescued. Thus, by an appeal to physical conformation, we were able to date the mental defect, and to save the reputation of a medical brother from undeserved opprobrium.

It will be within your remembrances that this town was some years since greatly moved by a sensational trial before a Master in Lunacy, in which an attempt was made to save from himself a youth with an honored name. Liberty of the Subject was the popular cry, and after conflicting evidence, the popular will prevailed, and the free agent went rapidly to his doom. The counsel for the defence, in specious terms, said that the mistake about this young man's imbecility arose from a defect in his mouth, by which imperfect speech resulted. I showed at that time—too late, however, to influence the verdict—that that was the most important admission that could be made—that, given any amount of mental obliquity, no stronger confirmatory proof could have been adduced of his imbecility than the physical defects of mouth to which this lisping speech was due. The sequel proved the congenital nature of the case.

No less valuable, however, is a study of the condition of the mouth as a means of prognosis in any given case. In children who exhibit any want of mental power, or present anomolous moral or intellectual symptoms, no more anxious question is suggested than that relating to the future of the case. The disposition of property and other family arrangements, depend a good deal on the answer which is given. We have learned by experience this important fact, that the child who has been born with defective intellect is more susceptible of improvement by physical and intellectual training than the child who has been born with full possession of his brain-power, and has afterwards been deprived thereof. In other words, that of two children who are the subjects of solicitude, other things being equal, there is greater probability of improvement for the patient with an ill-developed, than the one with a damaged, brain. Often it happens that a microcephalic idiot, about whom the inexperienced would entertain no sort of hope, will far outshine, under intellectual training, the fine, well-developed boy the membranes of whose brain have been the subject of inflammatory lesion, and about whose capillaries lymph has been inextricably effused. An appeal to the condition of the mouth is an important aid in determining whether lesion on which the mental weakness depends is of intra-uterine origin. In the event of the mouth being abnormal, it indicates a congenital origin; while if the mouth be well formed, and the teeth in a healthy condition, it would lead to the opinion that the calamity had occurred subsequently to embryonic life.

Of course, our judgment would be formed after a physical examination of every organ—of the condition of the ears and eyes—of the shape of the cranium; but what I want to enforce is, that most important information is derived from an examination of the mouth.

I have had this day brought to me a young girl of manifestly defective power, and the parents were extremely anxious to know whether it was a congenital case. Their anxiety was based partly on an unwillingness to own that there was on their side any hereditary tendency to mental disease—a sort of rivalry as to the purity of the two antecedent sources; but mainly also to ascertain with what degree of fear they must watch the development of their other children, and how it might affect the education of their sons, or the marriage prospects of their daughters. No study of the mental phenomena themselves would have enabled me to venture an opinion; but an examination into the totality of the physical conformation in general, and of the mouth in particular, enabled me to refer the calamity, with a considerable degree certainty, to a sun stroke in the tropics; to clear the other members of the family of the suspicion of insane proclivity, and to defend the purity of the rival stocks.

Let us now consider what are the conditions of the mouth associated with mental defect.

The lips are usually thick, the thickness being greatly more marked in the lower than in the upper one. In addition to the thickening, they are often straited, marked by transverse fissures; this character is more generally seen in a class of congenital idiots, which I have elsewhere described as Mongolian idiots, from their resemblance in physical character to the Mongolian race. Often the lips are deficient in muscular power, which interferes with their prehensile function, and which also induces a tendency for the saliva to run over the chin. The mucous membrane is extremely liable to chronic inflammation, and ulceration is produced by the slightest pressure against prominent or uneven teeth. The glands of the mucous membrane of the mouth generally, as well as the salivary glands, are usually hypertrophied; and this is another factor in the production of stammering.

There is a marked postponement in the evolution of the first teeth. Looking over my notes of a very large number of cases, I find that the first dentition is almost invariably postponed. The ease with which dentition is affected varies; sometimes the teeth are cut so easily that no disturbance to the general health is observed; in others it is the period at which violent convulsive attacks are developed, imperilling greatly the feeble mental endowment of the child. Contrary to the law that tissues which are slowly formed are the more persistent, these primary teeth have a more temporary existence than usual. They are frequently dark, speedily become carious, and of stunted growth, often the more stunted from the incessant grinding of the teeth, which is so frequent during the infantile life of the children. I have often been curious to ascertain the cause of this grinding. In most cases it appears to be a kind of automatic movement, not depending on the direct influence of the will; one of those rythmic movements of which there are several amongst children of this class. In others it would appear to be purposely developed to produce a monotonous sound, which imparts pleasure to the feeble-minded. Not only, however, are the primary teeth ill-developed; they are often irregularly developed as to sequence. Nothing but disorder is noticed in their succession.

Just as the evolution of the temporary teeth is accompanied by cataclysmic effects, like phenomena not unfrequently accompany the evolution of the permanent teeth. The epileptiform convulsion which accompany the first, not unfrequently, after a long interlude, and a recurrence at puberty. I have now under my observation a boy whose first teething was marked by paroxysmal phenomena of the most violent kind, which ceased when the physiological effort was over. He has now arrived at the period of second dentition, and the evolution of every tooth is accompanied by well-marked epileptic attacks, and corresponding decadence in his stunted mental development.

The evolution of the second teeth is frequently postponed; and there is slight irregularity in the sequence of their development. A marked character of the teeth is their irregularity as to position. They are often crowded—so crowded as to present their sides instead of their anterior surfaces. They are often arranged on different planes. The canine teeth are frequently unduly prominent, and a marked sulcus is sometimes seen between the incisors and canines, with prominence of the incisors. The teeth themselves present very frequently a honeycombed appearance, from an absence of continuity in the enamel, and they undergo speedy decay. Nothing is more marked than the tempo rary nature of the so-called permanent teeth.

It has been a matter of considerable interest to me to ascertain how frequently the syphilitic teeth, so well described by my friend and colleague, Mr. Hutchinson, were to be met with among the feeble-minded; but the result of my discovery has been to discover very few among them who were in this way indicated to be the subjects of congenital syphilis. Very few have had syphilitic teeth; but where I did discover them, I always had confirmatory evidence of the syphilitic history of the case, and the condition of the teeth was always associated with the chronic inflammation of the cornea, to which Mr. Hutchinson has called attention. I have, therefore, been led to the conclusion that syphilis is not by any means an important factor in the production of congenital mental disease. The honeycombed teeth are, I am persuaded, perfectly distinct from the syphilitic, and are manifestations of that grave perversion of nutrition which implicates in many cases every tissue in the body.

The tongue presents peculiarities worthy of notice. It is frequently unusually large. Its inordinate size generally arises from increase in length, and also from an absence of muscular tonicity. The surface is often curiously corrugated, presenting numerous fissures. The surface is rendered still more rough from hypertrophy of the papillæ. Not only is the tongue inconveniently large, it is but feeble under the influence of the will. There is a want of co-ordination in its muscles; so that not only is the more intelligen act of speaking performed awkardly when even there are ideas to be communicated, but the simple voluntary act of conveying the food to the posterior part of the mouth, where the reflex act of deglutition is excited, is effected with difficulty, and thus an almost instinctive act is rendered to some extent abortive.

Of most significant value, however, is the condition of the palate. I have made a very large number of careful measurements of the mouths of the congenitally feeble minded and of intelligent persons of the same age with the result of indicating, with some few exceptions, a markedly diminished width between the posterior bicuspids of the two sides. The exceptions were some few cases of Macrocephalic idiots, who had inordinately large crania, depending in some cases on hypertrophy of the brain, or more frequently on chronic hydrocephalus. In these exceptional cases the palates were as widely in excess as usually they are less than the normal width. One result, or rather one accompaniment, of this narrowing is the inordinate vaulting of the palate. The palate assumes a roof-like form. The vaulting is not simply apparent from the approximation of the teeth of the two sides, it is absolute—the line of junction between the palatal bones occupying a higher plane. Often there is an antero-posterior sulcus corresponding to the line of approximation of the two bones.

There is very frequently a deficiency in the posterior part of the hard palate, from a want of development of the palatal process of the maxillary bone, as well as absence of the palatal process of the palate bone. As a result of this defect, the false palate hangs down abnormally, and interferes with clear phonation. At an early period of my investigations I was prepared to find a large number of cases of cleft palate. This does not appear, however, to be a frequent defect—not more, according to my statistics, than five in one thousand cases. Bisection of the uvula occurred four times in one thousand, and absence of the uvula twice. The cause of this frequent excessive vaulting of the palate is not quite clear; it may possibly arise, as has been suggested, from arrest of development of the sphenoid bone, or defective growth of the vomer. It has been attributed by one writer to a cause which I think cannot be allowed, viz: sucking the thumb. This gentleman attributes idiocy entirely, or almost entirely, to fruitless sucking. The chain of events, according to his theory, is this:—The fruitless sucking gives rise to secretion of gastric juice in the stomach when it has no physiological use—that this acts as an irritant on the intestinal mucous membrane, giving rise to diarrhœa and disturbed nutrition, eventuating in convulsions and idiocy; and that the roofed-palate I have described is the physical result of the pressure of the child's thumb against the palate. Banish fruitless sucking, and idiocy would be unknown. I believe this to be a thoroughly mistaken notion. Idiots are not much more prone to fruitless sucking than other children, and I have examined the palates of several fruitless suckers among the sane without finding the palatal defects I have described. I think it will require no argument to prove that the defects I have described are developmental defects, and that they betoken a cause long anterior to the time when sucking the thumb is practiced, unless the habit be an intra-uterine one. The theory overlooks the whole bearing of hereditary influence, and of nervous shocks, and of physical illness to the pregnant woman, as potent causes of imbecility in the offspring, and negatives the idea of the congenital nature of the ailment.

There is one practical point with reference to the palatal defect which has some interest to me. One of the greatest difficulties we have, but one which, when overcome, brings the greatest *kudos*, is the teaching an idiot or imbecile to speak. We have to furnish him with a vocabulary, to prac tice him in tongue gymnastics, to build up for him a language, and to lessen the mutiny of the muscles of the tongue. When all this is in part accomplished, the arched roof of the palate is a great trouble. Imperfect speech still remains. Can nothing be done mechanically in this direction? Can the members of this Society tell us of any success they have had by diminishing the palatal cavity among the sane, that may augur hope for us with the insane? Often cases are met with where the palatal difficulty is just the hindrance to an improved imbecile mixing with the world, and taking his place with his fellows.

Among idiots and imbeciles there is often discovered a want of symmetry in the cranium. The plan which I adopt of taking the shape with a strip of lead shows this defect admirably, and enables me to sketch the precise deformity on paper. This want of symmetry also affects the bones of the jaw, leading to a great difference between the two halves of the maxillary bones. A very marked example of this kind was seen with me a short time since by my friend Mr. Ramsey, who advised the removal of some teeth to make room for the movements of the tongue. It was an excellent example also of the obtuseness to pain which is common among the feeble-minded. Often they are anxious to have their teeth extracted, as if it were a personal favor; they are flattered by the attention—rarely do they care for the pain which is inflicted—chloroform is for them a superfluity.

A want of symmetry is occasionally noticed in the palate where there is no very marked absence of symmetry elsewhere.

I might also mention anomalies of the tonsils and the fauces, but these would have but little interest to you.

The gums are extremely prone to recede from the teeth, and to become tumid, as in the scorbutic condition.

The masticatory process is always performed with difficulty. The carious condition of the teeth is one cause of this, but it is principally due to the imperfection with which any voluntary act is performed. The sense of taste is obtuse, no medicine, however nauseous, is refused, and without care many will eat with indifference the most offensive garbage.

To sum up, we have in the condition of the mouths of idiots important data for distinguishing between idiocy of a congenital and that of a non-congenital origin, and to base thereon a prognosis as to relative improvement by treatment. They are, the thickness of the lower lip, the delay of dentition, the chronic inflammation of the gums and buccal mucous membrane; the height, angularity, and want of symmetry of the palatal vault; the long corrugated and coarsely papulated tongue and the hypersecretion of saliva common to the congenital and non-congenital cases we meet with defect of mactication, of co-ordination of the muscles of the tongue, and the slight development of the faculty of speech.

I am anxious to hear from the members of this Society their own experience with regard to any feeble minded patients that may have come under their observation, and how far the picture I have drawn from nature contrasts with what they meet with in members of a sane community. It is quite possible that some of the characters I have noted may be met with sometimes among those who are doing their work in the world, and are among the bread-winners of society. My own experience leads me to consider that they are exceptional cases. May not deformities be indicative of a slightly degenerative process? When investigating cretinism in the valleys of Piedment, one is struck with the very gradual deterioration of the race. Some of the typical features of cretinism are met with among the grand-parents who are doing part of the world's work, and we have been able to trace the increased degenerative stages through the children to the effete grandchildren.

I have often had a lingering wish to measure and note the condition of the mouths of some of the progenitors of my patients, and trace—as I believe in some instances one could trace— the gradual deterioration of the species till it culminated in congenital imbecility.

That is just the link that I have not been able to forge. I know no men more likely than those I have the honor to address to-night to be able to fill up, from their own experience, the short comings of my own.—*Transactions of Odontological Society of Great Britain.*

